# Evaluation of Anthropometric Measurements of 17,693 Newborns: Have Percentile Cut-Off Values Changed?

**DOI:** 10.3390/children12050644

**Published:** 2025-05-16

**Authors:** Nursu Kara, Didem Arman, Adem Gül, Kudret Ebru Erol, Serdar Cömert

**Affiliations:** Department of Pediatrics, Division of Neonatology, University of Health Sciences Istanbul Research and Training Hospital, Istanbul 34025, Turkey; didem.arman@sbu.edu.tr (D.A.); adem.gul@saglik.gov.tr (A.G.); kudretebru.ozcan@saglik.gov.tr (K.E.E.); serdar.comert@sbu.edu.tr (S.C.)

**Keywords:** newborn, anthropometry, SGA, LGA, cut-off

## Abstract

Objective: The aim of our study was to develop current local anthropometric measurement percentiles for newborns and to compare these values with national and international growth chart percentiles. Methods: This retrospective cross-sectional study evaluated the birth records of 17,693 infants born between 24 and 42 weeks of gestation at the Health Sciences University Istanbul Training and Research Hospital between January 2018 and December 2023. The following data were collected from the birth records: type of delivery, gender, gestational week, birth weight, birth length, head circumference, and the nationality of the infants. Percentile charts for weight, length, and head circumference were generated according to gender and gestational week. The 10th, 50th, and 90th percentiles of the local anthropometric measurement percentiles were compared with the national and the international growth charts. Results: The anthropometric measurements of 17,693 newborns were evaluated in this study. Of the included infants, 9589 (54.2%) were born by normal spontaneous delivery and 8104 (45.8%) by cesarean section. A total of 4955 (28%) of the infants were preterm and 12,738 (72%) were term; 8700 (49.2%) were female and 8993 (50.8%) male. When compared by gender, it was observed that the birth weights of boys were higher than girls at all gestational weeks, but the lengths and head circumferences of both genders were similar. When our weight, length, and head circumference percentiles by gestational week were compared with the Fenton growth charts, we found that our babies had higher average values in all percentiles. When compared with national growth charts, the weight, length, and head circumference measurements of our girls and boys were higher, especially under 38 weeks, and they had similar anthropometric measurements from 38 weeks onwards. When compared with the Fenton growth chart, the ranges of difference from the current values used in each week of pregnancy for the SGA cut-off values for girls and boys were found to be 30–290 g and 30–230 g, respectively, and those for the LGA cut-off values for girls and boys were 80–300 g and 95–230 g, respectively. Conclusions: Our study reveals the current birth weight, length, and head circumference percentile values in infants, including a large number of infants in our region. Notably, the generated regional growth curves differ from existing international standards, which may have significant implications for the accurate diagnosis and follow-up of SGA and LGA infants. We suppose that our current national data can serve as a valuable reference for future multicenter studies involving larger populations and contribute to the optimal assessment of growth parameters in pediatric health surveillance.

## 1. Introduction

Monitoring growth, a crucial aspect of newborn and infant care, is of the utmost importance for both term and preterm infants. Evaluating anthropometric measurements such as body weight, length, and head circumference using reference growth charts provides valuable information regarding fetal and postnatal growth, perinatal morbidity and mortality, and developmental delays [1,2]. Birth weight and its alignment with gestational age are the most significant risk determinants for mortality and morbidity. Identifying infants born small for gestational age (SGA) or large for gestational age (LGA) is crucial for anticipating and managing potential short-term and long-term complications [3,4,5,6].

Normal fetal growth depends on the complex interaction of the fetal, placental, and maternal units. In addition to various maternal factors, environmental factors and ethnic and genetic characteristics also influence fetal growth. In essence, the primary determinant of growth is the infant’s own genetic potential and the extent to which this potential is affected by external factors. At this point, it is thought that evaluations made with universal standard curves, considering environmental and genetic conditions, may be misleading. The question of which reference curve should be used for accurate assessment is still debated. Currently, the most widely used curve in growth monitoring is the Fenton growth curve [7]. In addition to universal growth charts such as Intergrowth-21 and that of the WHO, some countries also have their own local growth charts [8,9,10,11]. Since there may be differences between standard anthropometric measurements in different populations, the ideal approach is to develop national growth charts that reflect the genetic characteristics of each society and to make evaluations in this context.

In our country, studies have been conducted in different regions, including term and preterm infants according to gestational week, covering different years and numbers of infants [12,13,14,15,16]. There are some limitations in these studies, such as the use of small samples and the inability to generate separate curves according to gender. Based on these facts, the aim of our study was to develop current, local growth percentiles based on gestational age and gender by recording the birth weight, length, and head circumference measurements of newborn infants born in our hospital, and to compare these values with national and international growth charts.

## 2. Materials and Methods

Our study was a retrospective cross-sectional study evaluating the birth records of all newborn infants born between 24 and 42 weeks of gestation at the Health Sciences University Istanbul Training and Research Hospital between January 2018 and December 2023. The following data were recorded from the birth records: type of delivery, sex, gestational week, birth weight, length, head circumference, and the nationality of the infants.

Gestational ages were determined by obstetricians or trained nurses in the delivery room based on the last menstrual period. If gestational age was not determined properly, it was evaluated by using Modified Ballard score in the nursery. Birth weight was recorded by a nurse in the delivery room using a gram-sensitive electronic scale (Medical Research Council, London, UK); length was measured using an infantometer; and head circumference was measured by the same person using a non-stretchable measuring tape from the widest occipitofrontal points.

Infants with severe congenital anomalies incompatible with life, those diagnosed with intrauterine growth restriction due to a fetal and/or maternal cause, those who were not singleton births, those whose gestational week could not be determined according to the last menstrual period, and those with missing data for weight, length, or head circumference measurements were excluded from the study. Based on these data, weight, length, and head circumference percentile curves were developed according to gender and gestational week. An intrauterine growth-restricted (IUGR) infant was defined as an infant with antenatal measurements below the 3rd percentile with an abnormal umbilical artery blood flow [17,18,19]. Infants below the 10th percentile for gestational age and gender were defined as SGA, and those above the 90th percentile were defined as LGA. The 10th, 50th, and 90th percentiles of the curves were compared with the national curves of Kurtoğlu et al. [15] and the growth curves developed by Fenton TR et al [7].

## 3. Statistical Analysis

Percentile curves of all our measurements were plotted with the SPSS Version 11 program, and extreme values were removed by excluding those below the 3rd percentile and those above the 97th percentile to try to homogenize the sample group. The Lambda–Mu–Sigma (LMS) method was used for the correction process. The curves were corrected using the LMS Chart Maker Pro version 2.3 software program (Medical Research Council, London, UK). The LMS method is based on the assumption that irregularities in the distribution (skewness) can be corrected by power transformation. The most appropriate “exponent” number for the “Box–Cox transformation” to be applied to normalize the distribution was calculated separately for each age group, and the trend of the distribution was summarized in the form of a curve (L). The mean value (M) and the coefficient of variation (S) were calculated in the same way. In our study, the curves were corrected as cubic splines using “penalized likelihood” with non-linear regression, and the limits of the correction were determined according to the equivalent degrees of freedom. The LMS v.5.1 program was used for these operations. Percentile curves (3, 5, 10, 25, 50, 75, 85, 90, 95, 97) were generated for each parameter from the obtained data.

## 4. Results

The total number of births in our hospital between January 2018 and December 2023 was 18,260. The anthropometric measurements of 17,693 newborns were used in generating the percentile values and graphs. Of the included infants, 9589 (54.2%) were born by normal spontaneous delivery and 8104 (45.8%) by cesarean section. A total of 4955 (28%) of the infants were preterm and 12,738 (72%) were term; 8700 (49.2%) were girls and 8993 (50.8%) were boys (Table 1).

The distributions of weight, length, and head circumference percentiles by gestational week for the female and male genders are shown in Table 2, Table 3 and Table 4, respectively.

**Figure 1:**
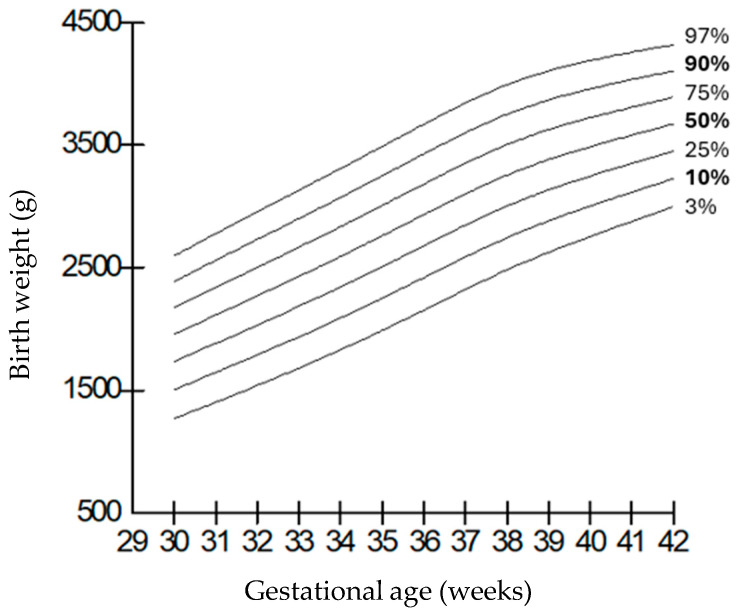
Birth weight percentiles for males according to gestational age.

**Figure 2:**
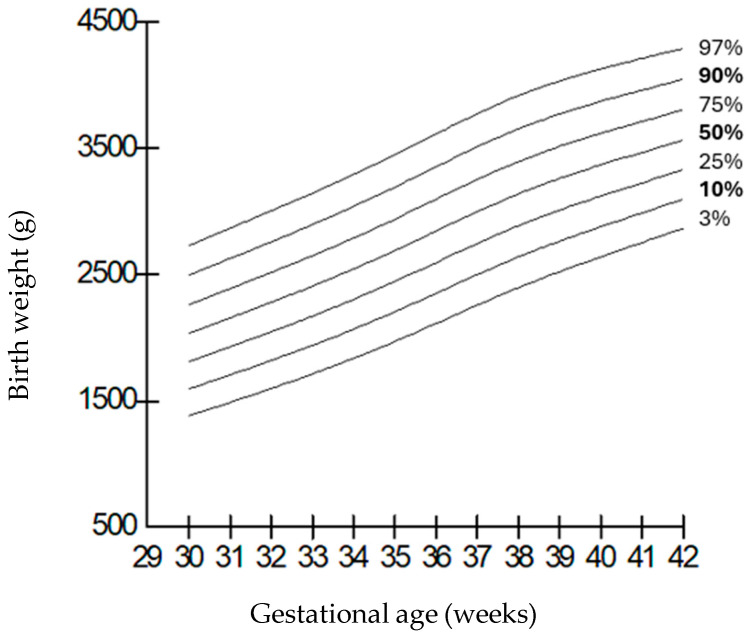
Birth weight percentiles for females according to gestational age.

**Figure 3:**
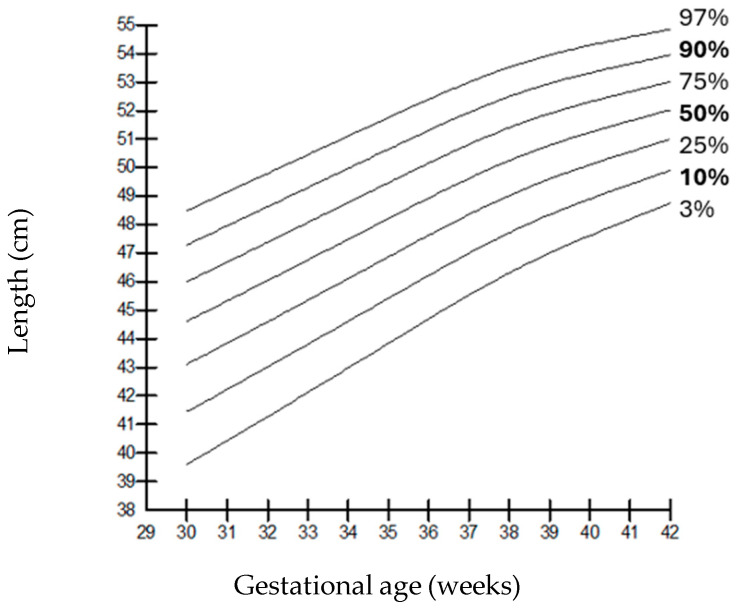
Length percentiles for males according to gestational age.

**Figure 4:**
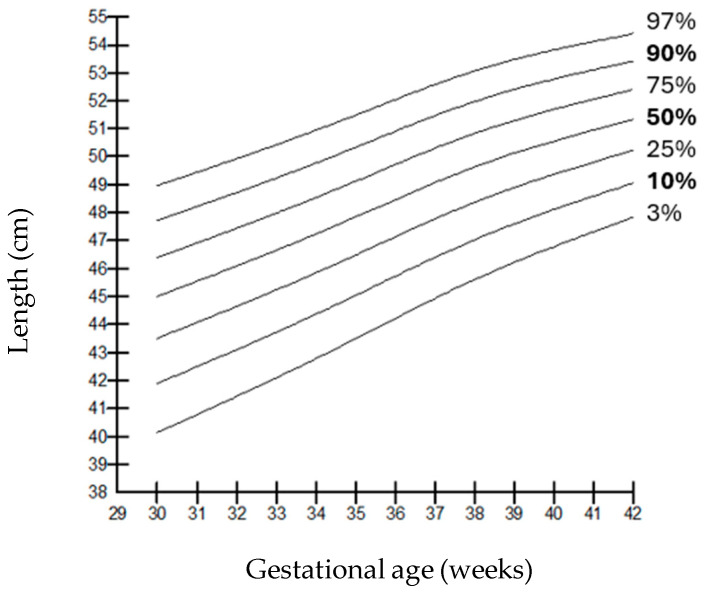
Length percentiles for females according to gestational age.

**Figure 5:**
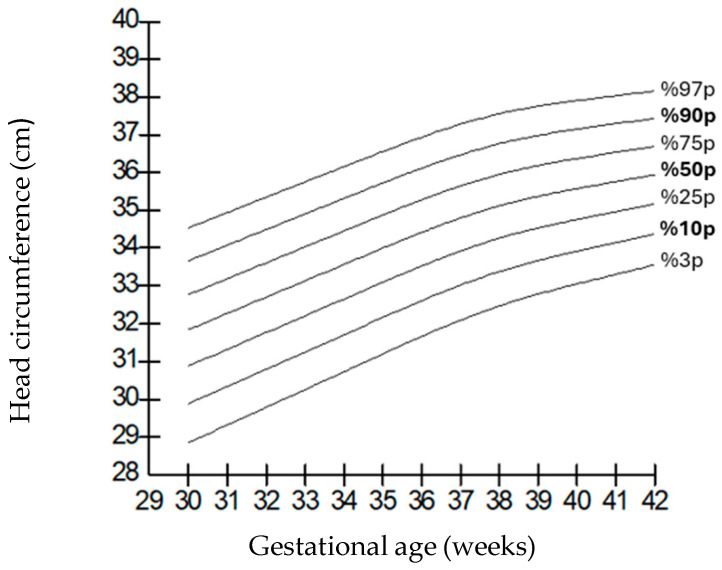
Head circumference percentiles for males according to gestational age.

**Figure 6:**
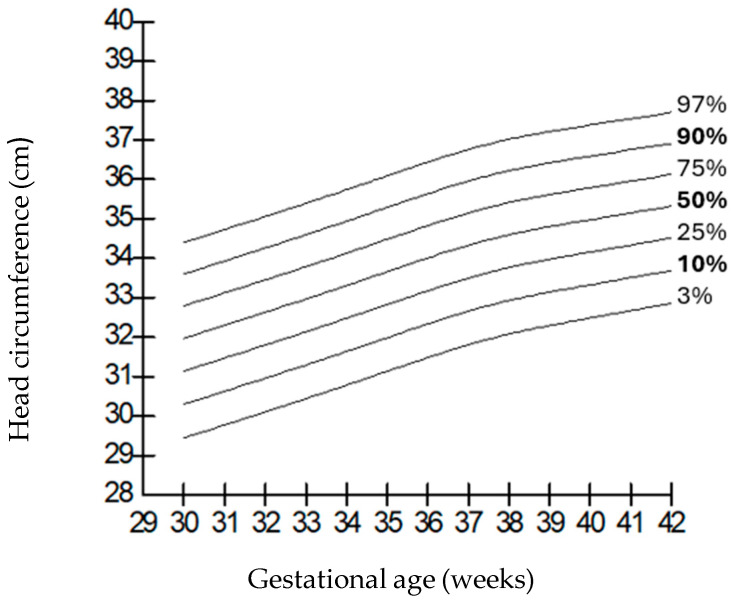
Head circumference percentiles for females according to gestational age.

When compared by gender, it was observed that the weights of boys were higher than those of girls at all gestational weeks, but the length and head circumferences of both genders were similar. Growth charts according to gestational week and gender-specific weight, length, and head circumference measurements of the infants born in our hospital are shown in Figure 1, Figure 2, Figure 3, Figure 4, Figure 5 and Figure 6.

**Figure 7:**
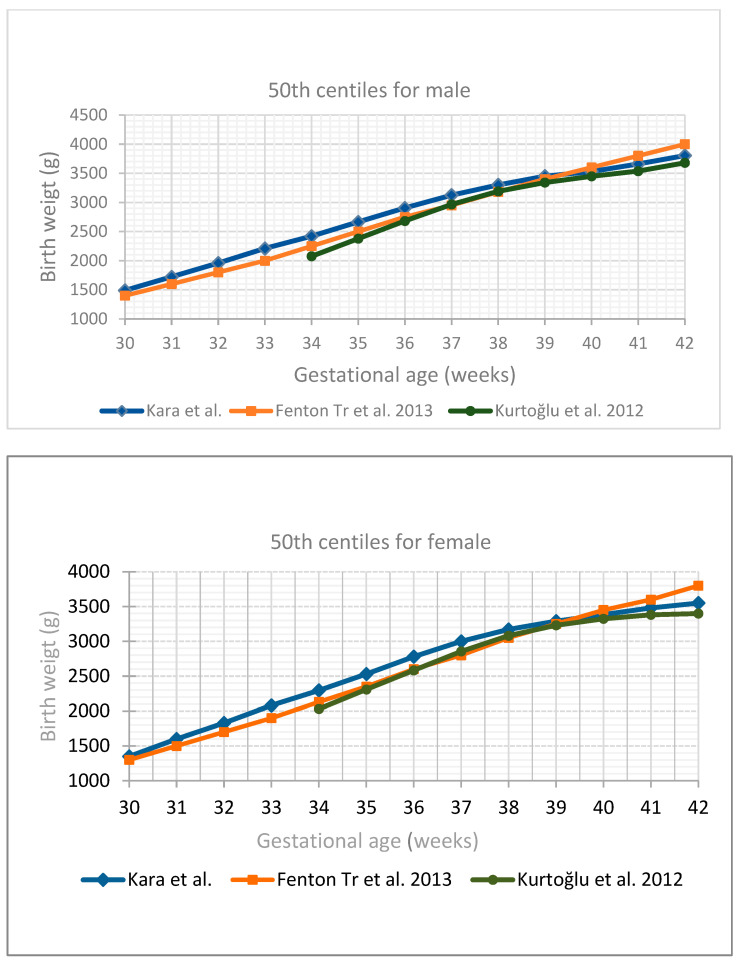
Comparison of 50th centiles of our study, Kurtoğlu et al. [15], and Fenton Tr et al. [7] growth charts for birth weight.

The comparison of the 50th percentile for gender and gestational week of all measurements obtained with the curves of Kurtoğlu S et al. [15] and the Fenton growth charts is shown in Figure 7.

When compared with the Fenton growth chart, the ranges of difference from the current values used in each week of pregnancy in SGA cut-off values for girls and boys were found to be 30–290 g and 30–230 g, respectively, and those for the LGA cut-off values for girls and boys were 80–300 g and 95–230 g, respectively.

## 5. Discussion

In our study, while the length and head circumference measurements of girls and boys were similar at all gestational weeks, the boys weighed more than the girls. When we compared the weight, length, and head circumference percentiles by gestational week for girls and boys with the Fenton international growth charts, we found that our babies had higher average values at all percentiles. When compared with the national growth charts of Kurtoğlu et al. [15], we found that the weight, length, and head circumference measurements of the girls and boys in our study were higher, especially under 38 weeks, and that they had similar anthropometric measurements from 38 weeks onwards. We found that the current SGA and LGA cut-off values that we determined were different for each week of pregnancy from those currently in use.

The World Health Organization (WHO) emphasizes the importance of monitoring the growth and development processes of children in child health follow-ups for the early detection of possible health problems and the implementation of necessary interventions [9]. The WHO also recommends the use of standard growth charts for the evaluation of growth and the monitoring of growth rate. The standard growth charts developed are an important reflection of the socioeconomic status of the society to which the selected infants belong, the level of access to health services, and nutritional data [10]. In addition, the fact that the reference group from which the standard values are obtained is from the same ethnic background and geographical region will minimize the errors that may occur during comparisons. At this point, even though universal growth standards have been determined, these standards may not be the most appropriate evaluation indicator for every society. This situation may reflect the need for societies to generate their own national or regional growth charts for their own babies.

When we compared the weight, length, and head circumference percentiles by gestational week for girls and boys with the Fenton growth charts, which we commonly use in the evaluation of growth in our country and clinic, we found that our babies had higher average values in all percentiles [7]. Since they reflect fetal and neonatal growth patterns in Turkish infants, we also compared our data with the growth charts developed by Kurtoğlu and Atıcı et al., which were conducted in our country and based on a population with similar geographic, ethnic, and socioeconomic characteristics. When we compared our results with the growth charts developed by Kurtoğlu et al., we found that the weight, length, and head circumference measurements of the girls and boys in our study were higher, especially under 38 weeks, and that they had similar anthropometric measurements from 38 weeks onwards [15]. When compared with the study by Atıcı et al., in which gender-specific data were shared from the 37th gestational week onwards, we found that our means and SGA and LGA cut-off values were higher for both genders between 37 and 42 weeks [14]. In the study conducted by Salihoğlu et al. in our country, we saw that especially the SGA cut-off values were significantly lower in both genders compared to our study. LGA cut-off values, on the other hand, were similar until the 37th gestational week, while they were found to be higher in our study in girls and boys after 37 weeks [16].

When we evaluated the anthropometric measurements in terms of gender, we found that, while the length and head circumference measurements of girls and boys were similar at all gestational weeks in our study, the weights of the boys were higher than the girls. There are many published studies showing that anthropometric measurements at birth differ between girls and boys depending on gender [15,20,21]. In their studies comparing the 10th, 50th, and 90th percentile weight, length, and head circumference values of newborns, Kurtoğlu et al. reported that male children had higher weights and lengths, and that their head circumference measurements were also higher than girls. In the same study, they found the difference between girls and boys to be an average of 200 g for weight, 0.8 cm for height, and 0.6 cm for head circumference [15]. In a study by Pawlus et al., from Poland, it was stated that all the anthropometric measurements of male infants were found to be higher than those of female ones [22]. These findings, along with our results, support the necessity of creating sex-specific percentile charts to ensure the accurate growth assessment and classification of newborns, given the differences in anthropometric measurements between males and females.

It is obvious that the use of national curves and percentile values is important in terms of detecting SGA and LGA infants, who are in the high-risk group [4,5,23,24,25]. The easiest and most reliable method that can be used to predict these risks is the evaluation of the appropriateness of birth weight for gestational age according to growth charts. Lubchenco growth charts have been widely used for many years around the world to define SGA, LGA, and AGA infants [26]. Studies comparing their own generated curves with Lubchenco growth curves have been reported. However, since it is thought that these historically important curves cannot fully reflect the demographic and health characteristics of today’s populations, it is thought that growth curves need to be updated. As a result of the updates made based on these curves, the most frequently used reference curves today are the Fenton growth curves, which were updated to be gender-specific in 2013 [3]. When we compared the values we obtained in our study with the Fenton growth charts, we found that the SGA and LGA cut-off values were different for each week of pregnancy. Previous studies have reported that the parameters contributing to this difference include maternal weight gain, BMI, paternal birth weight, socioeconomic status, antenatal follow-up, and genetic factors [27]. According to 2018 Turkish Statistical Institute statistics [28], approximately 90% of mothers in Istanbul received optimum antenatal care before birth, underscoring the positive influence of this approach on neonatal anthropometric measurements, which may possibly be an explanation for the differences. Since our study was designed as a retrospective study, we were unable to access these data regarding other confounding factors. When we evaluated our newborns with the regional curve percentiles, we found that the number of infants defined as SGA was higher and the number of infants defined as LGA was lower. This situation can lead to SGA infants being defined as AGA and experiencing problems in receiving the care they need. In addition, since AGA infants are defined as LGA, they may be exposed to unnecessary intensive care monitoring, which may lead to the misuse of intensive care resources.

The fact that our study included the largest infant group in our country and defined updated, current 10th and 90th percentile cut-off values makes our study valuable. However, being single-centered is one of the limitations of our study. Due to the retrospective nature of our study, the inability to access maternal and paternal data represents a limitation. Another limitation of our study is that the number of <30 week premature newborns was not sufficient, and, therefore, growth percentiles at these weeks could not be defined.

As a result, our study reveals the current birth weight, length, and head circumference percentile values in infants in our region, based on a large number of infants. Notably, the generated regional growth curves differ from the existing international standards, which may have significant implications for the accurate diagnosis and follow-up of SGA and LGA infants. We suppose that our current national data can serve as a valuable reference for future multicenter studies involving larger populations and contribute to the optimal assessment of growth parameters in pediatric health surveillance.

## Figures and Tables

**Table 1:** Demographic characteristics of study population.

		*n* = 17,693
Gender (*n*)/(%)	Female	8700 (49.2%)
	Male	8993 (50.8%)
Route of delivery (*n*)/(%)	NSVD	9589 (54.2%)
	C/S	8104 (45.8%)
APGAR score (min.–max./median)	1st min	1–9 (8)
	5th min	5–10 (9)
Gestational week *		38.21 ± 5.2
Birth weight (g) *		3188.49 ± 579.62
Birth length (cm) *		49.74 ± 5.7
Head circumference (cm) *		34.90 ± 2.6

NSVD: normal spontaneous vaginal delivery: C/S: cesarean. * (mean ± SD).

**Table 2:** Birth weight percentages of male and female neonates according to gestational age.

Birth Weight Percentages (g)															
Gestational Age	Gender	L	M	S	3rd	5th	10th	15th	25th	50th	75th	85th	90th	95th	97th
24–26	Female	−0.319	790	0.186	435	545	595	600	650	790	900	930	935	946	1025
	Male	0.079	715	0.34	450	475	550	600	655	715	895	960	1005	1040	1240
27–29	Female	2.832	1000	0.374	515	590	650	680	817	1000	1145	1235	1510	1600	1800
	Male	2.943	1200	0.332	650	735	865	900	1040	1200	1345	1415	1500	1690	1785
30–31	Female	1.776	1725	0.342	930	1010	1130	1285	1410	1725	1800	1900	2000	2200	2300
	Male	0.815	1520	0.216	850	990	1115	1240	1330	1520	1740	1870	1985	2075	2175
32	Female	0.781	1830	0.230	1170	1245	1410	1470	1580	1830	2150	2350	2400	2630	2700
	Male	1.245	1830	0.285	1120	1205	1385	1425	1585	1830	1970	2130	2240	2390	2540
33	Female	1.153	2080	0.212	1490	1545	1680	1730	1910	2080	2350	2540	2660	2855	3010
	Male	0.029	2210	0.176	1490	1530	1620	1780	1930	2210	2490	2540	2640	2930	3000
34	Female	0.205	2285	0.163	1585	1680	1870	1960	2078	2285	2490	2560	2870	2990	3150
	Male	0.240	2390	0.157	1630	1700	1885	1960	2150	2390	2615	2805	2870	3060	3160
35	Female	0.446	2530	0.156	1870	1920	2090	2170	2330	2530	2825	2970	3100	3250	3360
	Male	0.112	2565	0.169	1780	1825	2050	2150	2310	2565	2840	2975	3070	3390	3480
36	Female	0.037	2805	0.151	1970	2080	2270	2355	2525	2805	3040	3180	3350	3485	3610
	Male	0.112	2565	0.169	1780	1825	2050	2150	2310	2565	2840	2975	3070	3390	3480
37	Female	0.148	3000	0.142	2190	2345	2490	2620	2750	3000	3235	3300	3590	3730	3800
	Male	0.273	3085	0.133	2350	2450	2630	2700	2830	3085	3340	3510	3645	3830	3940
38	Female	0.189	3170	0.127	2450	2540	2680	2790	2930	3170	3430	3590	3780	3840	3950
	Male	0.203	3300	0.122	2570	2690	2825	2920	3050	3300	3570	3730	3845	4000	4120
39	Female	0.080	3290	0.132	2500	2625	2760	2850	3010	3290	3580	3740	3900	3990	4100
	Male	−0.119	3450	0.119	2605	2720	2910	3028	3170	3450	3710	3860	3950	4080	4375
40	Female	−0.117	3380	0.127	2670	2745	2830	3015	3130	3380	3685	3830	3970	4140	4270
	Male	0.358	3530	0.126	2810,0	2880	3030	3140	3260	3530	3840	4030	4160	4380	4500
41	Female	0.136	3480	0.124	2725	2805	2995	3100	3225	3480	3790	4000	4100	4235	4420
	Male	−0.018	3660	0.123	2990	3055	3170	3270	3360	3660	3960	4130	4225	4415	4630
42	Female	0.233	3550	0.136	2845	2970	3025	3115	3375	3550	3890	4260	4270	4420	4585
	Male	0.792	3775	0.152	3132	3200	3280	3350	3490	3775	4165	4260	4450	4675	4890

L: lambda, skewness; M: mu, median; S: sigma, coefficient of variation.

**Table 3:** Birth length percentages of male and female neonates according to gestational age.

Birth Length Percentages (g)															
Gestational Age	Gender	L	M	S	3rd	5th	10th	15th	25th	50th	75th	85th	90th	95th	97th
24–26	Female	−0.529	32.0	0.120	25.2	26.7	27.1	28.0	29.3	32.0	34.8	35.0	37.0	38.0	38.0
	Male	−0.428	32.5	0.081	27.0	27.5	28.0	28.4	30.0	32.5	34.0	35.0	37.0	38.2	38.4
27–29	Female	1.543	35.0	0.106	29.5	30.0	32.0	32.0	33.0	35.0	36.0	38.3	39.7	41.0	42.7
	Male	1.544	36.0	0.093	32.0	32.7	33.0	34.0	35.0	36.0	38.0	40.1	41.7	43.4	46.9
30–31	Female	−0.188	42.0	0.106	32.6	33.0	36.1	38.7	39.8	41.0	43.0	44.4	45.0	45.4	46.0
	Male	−0.041	41.5	0.071	36.0	36.7	37.5	38.0	39.0	40.5	42.8	44.0	46.0	46.8	48.1
32	Female	1.146	43.0	0.084	35.7	38.6	39.0	39.4	41.0	43.0	44.0	46.0	47.4	48.0	48.4
	Male	0.185	43.0	0.081	37.0	37.5	38.5	40.0	41.0	43.0	45.0	46.0	47.0	48.0	50.1
33	Female	−0.331	45.0	0.070	38.0	40.0	41.0	42.0	44.0	44.5	47.0	48.0	48.4	50.8	51.0
	Male	−0.982	45.0	0.077	37.9	39.0	40.3	41.0	43.0	45.0	47.0	48.0	48.7	49.9	50.5
34	Female	−0.493	46.0	0.053	40.0	42.0	42.4	43.0	44.0	46.0	47.3	48.0	49.0	49.2	50.0
	Male	−0.930	47.0	0.061	40.0	41.0	42.0	44.0	45.0	47.0	48.0	49.0	49.0	50.7	51.0
35	Female	−0.399	47.5	0.048	42.0	43.0	43.8	45.0	46.0	47.5	49.0	49.4	50.0	51.0	51.5
	Male	−0.687	48.0	0.057	42.0	42.5	44.0	44.0	46.0	48.0	49.0	50.0	50.0	51.5	52.0
36	Female	−1.159	48.0	0.050	43.0	44.0	45.0	46.0	47.0	48.5	50.0	50.0	51.0	51.0	52.0
	Male	−0.213	49.0	0.043	45.0	45.2	46.0	47.0	48.0	49.0	50.0	51.0	51.0	52.0	53.0
37	Female	−1.362	49.0	0.045	45.0	46.0	46.0	47.0	48.2	49.5	50.0	51.0	52.0	52.0	53.0
	Male	−0.434	50.0	0.040	46.0	46.5	47.0	48.0	48.0	50.0	51.0	52.0	52.0	52.4	53.0
38	Female	−0.435	50.0	0.040	46.0	46.3	47.0	48.0	48.6	50.3	51.0	52.0	52.8	53.0	53.5
	Male	−0.078	50.0	0.038	47.0	47.3	48.0	48.0	49.0	50.0	52.0	52.0	53.0	53.0	54.0
39	Female	−0.658	50.0	0.040	46.3	47.0	48.0	48.4	49.0	51.0	51.4	52.4	53.0	53.5	54.0
	Male	−0.340	51.0	0.038	47.5	48.0	49.0	49.0	50.0	51.0	52.0	53.0	53.5	54.0	54.0
40	Female	−0.306	51.0	0.039	47.0	48.0	49.0	49.2	50.0	51.4	52.0	53.0	53.5	54.0	54.5
	Male	−0.835	51.0	0.040	48.0	49.0	49.0	49.5	50.0	51.0	52.5	53.0	54.0	54.5	55.0
41	Female	−0.096	51.0	0.036	47.2	48.4	50.0	50.4	51.0	52.4	52.6	53.0	54.0	54.5	55.2
	Male	−0.256	52.0	0.037	48.5	49.0	50.0	50.0	51.0	52.0	53.0	53.5	54.2	55.0	55.0
42	Female	0.382	51.0	0.044	47.6	49.8	51.0	51.2	51.8	53.2	53.6	54.0	54.5	55.2	55.8
	Male	−0.234	52.0	0.041	49.0	49.4	50.0	50.0	51.0	52.5	53.8	54.3	54.8	55.7	56.0

L: lambda, skewness, M: mu, median, S: sigma, coefficient of variation.

**Table 4:** Head circumference of male and female neonates according to gestational age.

Head Circumference Percentiles (cm)															
Gestational Age	Gender	L	M	S	3rd	5th	10th	15th	25th	50th	75th	85th	90th	95th	97th
24–26	Female	0.218	23.3	0.095	19.1	19.9	20.0	20.6	21.3	23.3	24.5	25.5	26.0	26.5	28.7
	Male	0.406	23.0	0.092	20.0	20.0	20.5	21.0	21.3	23.0	24.8	25.3	25.8	26.0	26.5
27–29	Female	1.285	25.8	0.100	23.6	23.8	24.0	24.4	24.8	25.8	26.9	28.0	29.3	32.0	33.1
	Male	1.179	27.0	0.085	24.0	23.7	24.2	25.0	25.3	27.0	28.0	29.0	29.0	32.0	33.0
30–31	Female	−0.430	30.0	0.085	26.6	26.8	26.9	27.0	27.2	27.6	31.0	31.4	32.0	33.0	33.8
	Male	−0.147	30.0	0.060	26.3	27.0	27.2	27.8	28.5	30.0	31.0	31.2	32.2	33.0	34.0
32	Female	0.231	30.0	0.059	27.8	28.0	28.2	28.4	29.0	29.8	31.4	32.0	32.3	33.4	34.6
	Male	0.278	31.0	0.073	27.0	27.3	28.0	28.3	29.0	31.0	32.9	33.0	33.8	34.5	35.0
33	Female	−1.915	32.0	0.067	28.4	29.0	29.2	30.0	31.0	31.4	32.8	33.0	33.4	34.8	35.0
	Male	−0.064	32.0	0.050	27.5	28.0	29.0	30.0	31.0	32.0	33.0	34.0	34.0	35.3	36.0
34	Female	2.655	32.0	0.074	29.0	29.2	30.0	30.8	31.6	32.1	33.0	34.0	34.4	35.1	36.0
	Male	−0.029	33.0	0.049	28.0	30.0	31.0	31.0	32.0	33.0	34.0	34.0	34.4	36.0	36.5
35	Female	−0.069	33.0	0.043	29.6	30.0	30.8	32.0	32.2	33.0	34.0	34.4	35.2	36.0	36.4
	Male	−0.367	34.0	0.046	30.0	31.0	32.0	32.0	33.0	34.0	35.0	35.0	35.0	36.5	36.9
36	Female	1.580	34.0	0.044	30.2	30.8	31.5	32.4	33.0	33.8	35.0	35.4	35.9	36.2	36.8
	Male	−0.172	34.0	0.042	31.0	32.0	33.0	33.0	33.5	34.0	35.0	36.0	36.2	37.0	37.5
37	Female	1.429	34.0	0.046	30.8	31.0	32.2	33.0	33.8	34.5	35.4	36.0	36.4	36.5	37.0
	Male	−0.445	35.0	0.041	32.0	33.0	33.0	33.5	34.0	35.0	36.0	36.0	36.5	37.4	37.8
38	Female	0.424	35.0	0.037	31.4	31.8	32.7	33.5	34.0	35.0	36.0	36.2	36.7	36.9	37.5
	Male	−0.175	35.0	0.037	33.0	33.0	34.0	34.0	34.0	35.0	36.0	36.9	37.0	37.8	38.0
39	Female	−0.014	35.0	0.041	32.0	32.4	33.2	33.8	34.2	35.5	36.2	36.7	36.9	37.0	37.8
	Male	−0.015	35.5	0.038	33.0	33.0	34.0	34.5	35.0	35.5	36.0	37.0	37.5	38.0	38.5
40	Female	3.440	35.0	0.046	32.6	32.8	33.7	34.0	34.6	35.8	36.6	36.9	37.2	37.4	38.0
	Male	1.469	36.0	0.040	33.0	34.0	34.0	34.0	35.0	36.0	36.0	37.0	37.8	38.5	39.0
41	Female	0.048	35.0	0.037	33.0	33.2	34.2	34.4	35.0	36.2	36.8	37.0	37.5	37.8	38.2
	Male	−1.240	36.0	0.039	33.0	34.0	34.0	35.0	35.0	36.0	37.0	37.0	38.0	38.0	39.5
42	Female	−1.104	35.0	0.042	33.4	33.7	34.7	34.8	35.4	36.4	37.0	37.5	37.8	38.0	38.8
	Male	0.111	36.0	0.035	34.0	34.0	34.4	35.0	35.0	36.0	37.0	38.0	38.5	39.0	40.0

## Data Availability

The raw data supporting the conclusions of this article will be made available by the authors on request.
